# Study on the correlation between lifestyle and negative conversion time in patients diagnosed with coronavirus disease (COVID-19): a retrospective cohort study

**DOI:** 10.1186/s12889-023-17163-9

**Published:** 2023-12-04

**Authors:** Nan Li, Chenbing Liu, Lihong Qiu, Chao Shen, Feng Zhang, Zhangfan Lu, Menghao Zhou, Di Sheng, Zhong Liu

**Affiliations:** Health Management Center, The First Affiliated Hospital of Zhejiang University School of Medicine, No. 79 Qingchun Road, Hangzhou, Shangcheng District China

**Keywords:** COVID-19, Lifestyle, Negative conversion time, Restricted cubic spline models

## Abstract

**Background:**

As of early December 2022, China eased the coronavirus disease (COVID-19) restriction, affecting over 80% of the country’s population and posing a severe threat to public health. Previous studies mostly focused factors on the severity/mortality rate of hospitalized COVID-19 patients, but limited studies explored factors associated with virus-negative conversion, particularly lifestyles. Therefore, the aim of our study was to analyze the correlation between lifestyle factors and the negative conversion time in COVID-19 patients.

**Methods:**

We recruited individuals aged 18 years or older who had a clear time record for both the diagnosis and negative conversion of COVID-19 and completed the electronic questionnaire with no missing data. Dietary data collected from the questionnaire was analyzed using exploratory factor analysis to establish dietary patterns. Age segmentation was performed using restricted cubic spline (RCS) plots. The association between lifestyle factors and the time to negative conversion in different age groups, was assessed using Kaplan-Meier plots and Cox regression analysis.

**Result:**

Out of 514 participants, all achieved viral negative conversion within a median time of 11 days. Based on nutrient intake, we identified four dietary patterns. The relationship between age and negative conversion rate, as depicted by RCS plots, exhibited an inverted “U” shape. We categorized age into three segments: <35 years, 35–45 years, and ≥ 45 years. For individuals under 35, our study indicated that a higher protein intake was linked to a faster recovery among COVID-19 patients, while medical staff or those receiving prescription treatments exhibited a slower recovery rate (*P* < 0.05). The 35 ~ 45 age group showed that adequate sleep and physical exercise were associated with a shorter time to negative conversion, whereas southern regions and a higher intake of carbohydrates were related with a longer conversion time (*P* < 0.05). Among individuals aged ≥ 45 years, the negative conversion time was primarily associated with physical exercise and being a medical staff member(*P* < 0.05).

**Conclusion:**

Our research suggests that adequate sleep, physical exercise and a higher protein intake can help alleviate COVID-19 symptoms, while a higher level of carbohydrates intake may hinder recovery from COVID-19.

## Background

Coronavirus disease (COVID-19), a Public Health Emergency of International Concern, caused by severe acute respiratory syndrome coronavirus 2 (SARS-CoV-2), was first reported in China at the end of 2019 [1, 2]. Since the first case was reported, the pandemic has rapidly spread worldwide, affecting over 181 countries and billions of people [3]. The common symptoms include fever, cough, and fatigue, and in some cases, individuals may experience headaches, diarrhea, sore throat, as well as olfactory and taste disturbances [4, 5]. Some studies indicated that COVID-19 can cause gastrointestinal tract and kidney damage, and even increase the incidence rate of osteoporotic hip fractures [6, 7]. More critically, COVID-19 infection can induce the onset of cancer and worsen the course of diagnosed cancer [8, 9]. On December 7, 2022, China’s epidemic prevention and control policy was fully lifted, leading to a significant increase in the number of COVID-19 cases within the country. Therefore, there is an urgent need to clarify the factors associated with COVID-19 to mitigate its consequences.

Previous studies have focused on the factors that affect the severity and mortality of hospitalized COVID-19 patients, including gender, age, lifestyle, obesity, hypertension, and clinical indicators [10–15]. However, there are few studies on the factors influencing the achievement of virus negative conversion. A cohort study proved that a higher level of plasma 25 (OH) D was independently associated with a shorter time to nucleic acid negative conversion for COVID-19 patients [16]. Furthermore, Lang LW et al [17] have identified low albumin levels especially when below 38 g/L, can be a risk factor for delayed negative conversion in COVID-19 patients. Another study releveled that neutrophil-to-lymphocyte ratio (NLR) is a simple and useful prognostic factor for predicting negative conversion time of nucleic acid in nonsevere COVID-19 patients with Omicron variant [18]. However, the relationship between lifestyle factors and the duration of negative transition in confirmed COVID-19 patients remains unknown. Therefore, the aim of our study was to investigate the influence of lifestyle factors including smoking, drinking, dietary patterns, and sleeping hours, on the time it takes for COVID-19 patients to achieve negative conversion and to provide valuable insights and effective guidance to help individuals prevent severe COVID-19 outcomes.

## Materials and methods

### Research settings

The study was conducted by the Health Management Center of the First Affiliated Hospital of Zhejiang University School of Medicine from January 12, 2023 to January 27, 2023. To collect data on COVID-19, we developed an electronic questionnaire through an online survey system (www.wjx.com). The questionnaire was disseminated to participants via WeChat Moments. As the most popular social media platform in China, WeChat Moments enables a larger group to participate in the questionnaires. This study was approved by the local ethics committee of the First Affiliated Hospital of Zhejiang University School of Medicine. All subjects had thoroughly understood the research requirements. By returning the completed questionnaires, participants provided implied informed consent. All responses were kept anonymous and participants had the option to withdraw their participation at any time before submitting the final questionnaire. This study was completed in accordance with the Helsinki Declaration.

### The questionnaire

The electronic questionnaire mainly involved four aspects: [1] demographic characteristics: date of birth, sex, marital status, habitation, and medical staff; [2] lifestyle: smoking, drinking, physical exercise, bedtime, sleeping hours, and dietary patterns. Based on the dietary data from the 1992 China Health and Nutrition Survey and the current dietary patterns of Chinese residents, we have classified and integrated the main food categories into 8 food categories: cereals and starch (such as rice, noodles, and various grains), meat and poultry, vegetables and fruits, seafood (including fish and other aquatic products), dairy products, eggs and related products, beans and legumes. For each food category, we recorded the frequency and daily or weekly intake; [3] underlying health conditions, including hypertension, diabetes, hyperlipidemia, chronic respiratory disease, chronic kidney disease, rheumatic disease, and cancers, etc. [4]. COVID-19 information: COVID-19 vaccination situation, whether or not infected with COVID-19, the date of infection of COVID-19 and the detection pathway, the treatment methods of COVID-19 infection, whether or not tested negative for COVID-19 and the exact date of testing negative.

### Study Population

​The study enrolled participants aged 18 years or older who completed the electronic questionnaire on COVID-19 between January 12 and January 27, 2023. A total of 1475 questionnaires were collected in this study. Participants who met the following exclusion criteria were excluded from the study:

1. those with missing or extreme outlier data.
2. individuals who were not infected with COVID-19 or did not meet the diagnostic criteria for COVID-19 after December 2022.
3. no clear time and evidence for negative conversion of COVID-19.

**Fig. 1:**
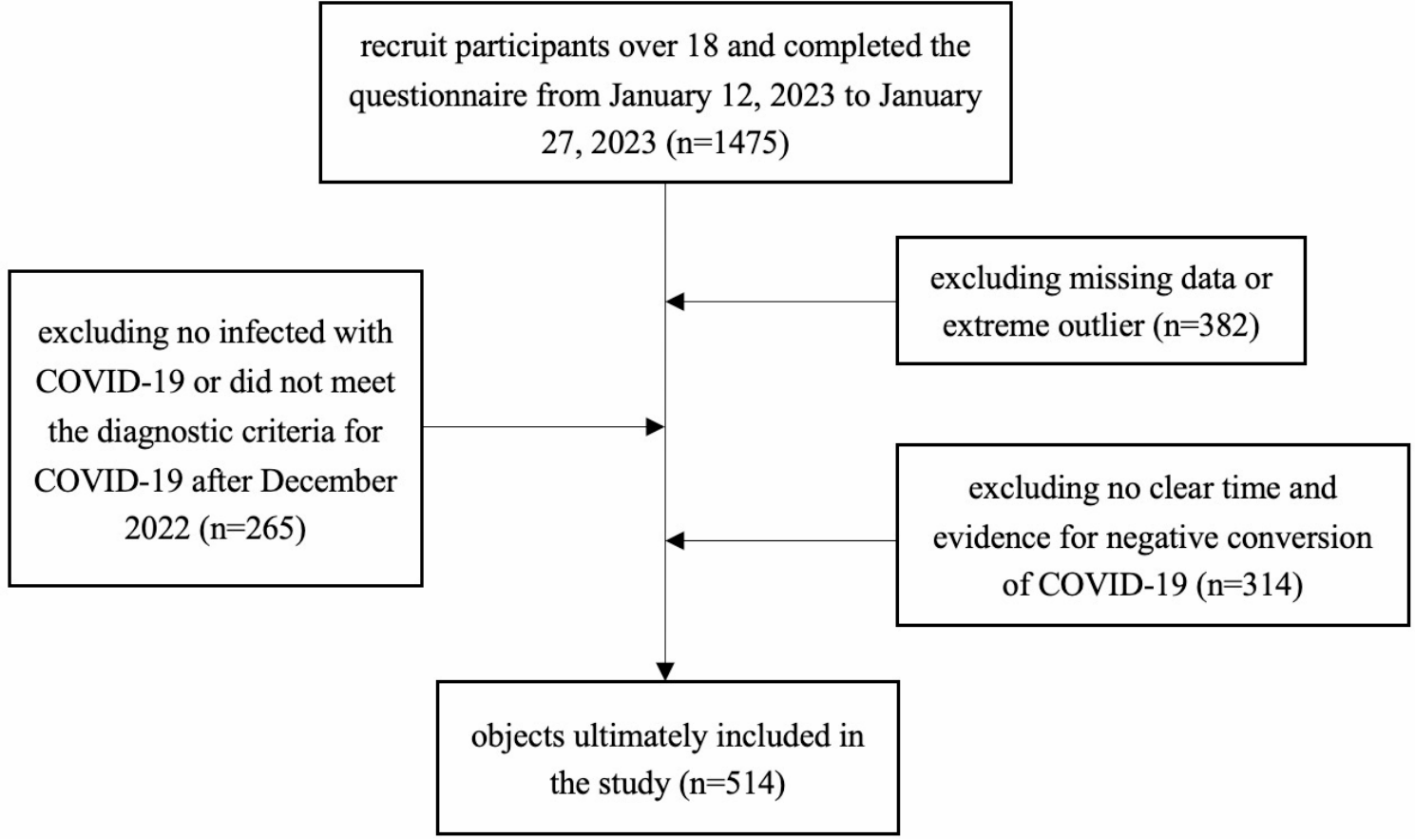
The flow chart of this study

Individuals who met both the inclusion and exclusion criteria were all included. A total of 514 subjects, including 126 males and 388 females, were included in the study. The flowchart of this study is shown in Fig. 1.

### Diagnostic criteria for diseases and related definitions

Diagnostic criteria of COVID-19 include individuals who exhibit clinical manifestations related to COVID-19 infection, and meet one or more of the following test results: [1] positive nucleic acid test for COVID-19; [2] positive COVID-19 antigen test; [3] positive COVID-19 isolation and culture; [4] a four-fold or higher increase in COVID-19 specific IgG antibody levels during the recovery phase compared to the acute phase [19]. The diagnostic criteria for negative conversion of COVID-19 are nucleic acid tests or other pathogenic and serological examinations negative on respiratory samples such as sputum and nasopharyngeal swabs.

Smoking was defined as the average consumption of at least 1 cigarette per day for a duration of ≥ 1 year or less than 1 year since smoking cessation. Quit smoking referred to individuals who had stopped smoking for at least 1 year. Never drinking was defined as not consuming alcohol in the past year or only consuming a small amount on special occasions. Drinking was considered for individuals who had consumed alcohol at least once a week within the past year. Quit drinking indicated individuals who used to drink regularly but had stopped for the past year. Physical exercise was defined as engaging in 20 min or more of leisure activity per day.

### Statistical analysis

The data were analyzed using R version 4.2.2 and IBM SPSS version 23.0. Non-normal distributed continuous variables were presented as median (P25, P75), and between-group comparisons were performed using the Mann–Whitney U test. Categorical variables were represented as the number of subjects (%), and between-group comparisons were conducted using the chi-square test. Four dietary patterns were identified using exploratory factor analysis. The scores for each dietary pattern were categorized into T1, T2 and T3 groups according to the tertiles, representing low to high scores. The association between age and COVID-19 negative conversion was evaluated using restricted cubic spline (RCS) models based on Cox proportional hazards models. Age was categorized into three segments based on the above results. Kaplan-Meier survival analysis and Cox regression analysis were used to analyze factors influencing the time to negative transition in COVID-19 patients among different age groups. Significance was defined as a *P* value < 0.05.

## Results

### Baseline characteristics of patients with COVID-19

**Fig. 2:**
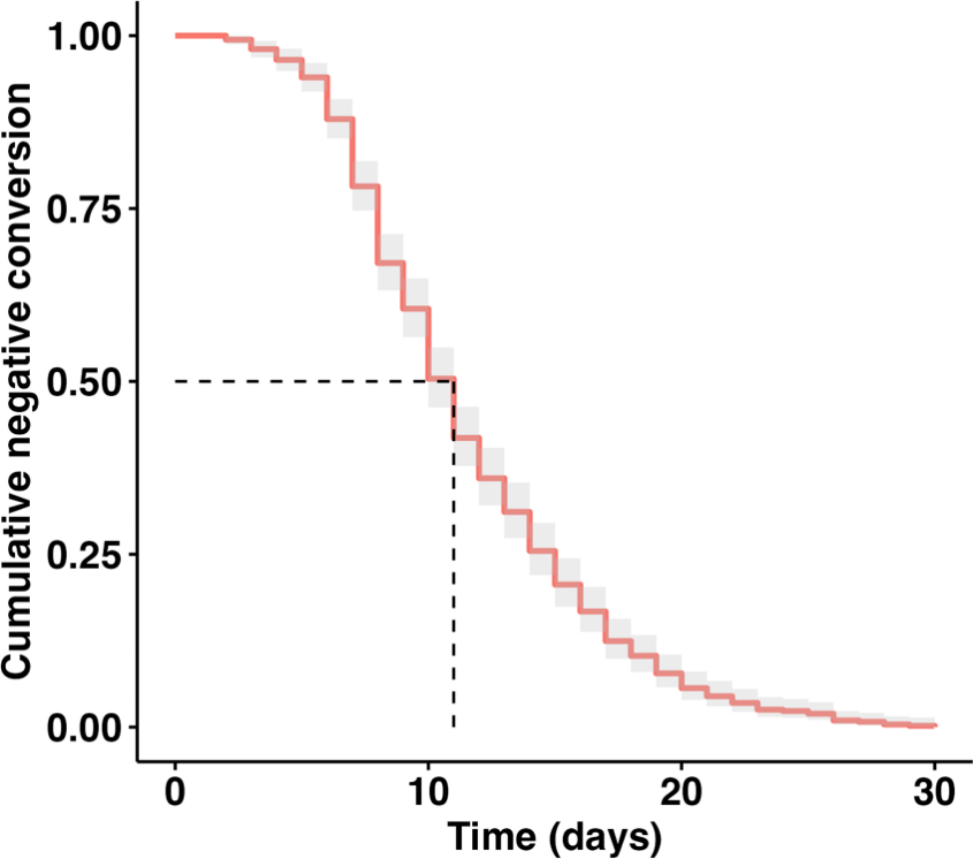
Negative conversion curves estimating the cumulative probability of negative conversion of COVID-19 patients

A total of 514 participants, comprising 126 males and 388 females, were included in our study. The median age was 35 years (ranged from 20 to 78 years), and only 8 patients (1.6%) were elderly (≥ 60 years). Out of 514 patients, 100 (19.46%) had underlying health conditions, including hypertension (8.0%, n = 41), diabetes (2.3%, n = 12), hyperlipidemia (8.4%, n = 43), chronic respiratory disease (1.2%, n = 6), chronic kidney disease (0.8%, n = 4), rheumatic disease (1.6%, n = 8), and cancers (1.4%, n = 7). Regarding vaccination, 8 (1.6%) patients had not received any COVID-19 vaccination, 5 (1.0%) had received only one dose, 45 (8.8%) had received two doses, 429 (83.5%) had received three doses, and 27 (5.3%) had received four doses. Importantly, there was no significant difference in the time of negative conversion among different vaccination groups (*H* = 1.436, *P* = 0.838). In addition, all patients included in the study achieved viral negative conversion. The time it took for all patients to achieve negative conversion raged from 2 to 30 days, with a median time of 11days (Fig. 2).

The population was categorized into two groups: group A (negative conversion time < 11 days) and group B (negative conversion time ≥11 days). Table 1 suggested that southern residents, medical staff, individuals with no physical exercise, those who had a later bedtime, and those with shorter sleeping hours showed a delayed negative conversion (*P* < 0.05). However, no significant associations were observed between the time of negative conversion and other factors such as age, sex, marital status, location, smoking, drinking, underlying health conditions, COVID-19 vaccination, and treatment methods.

**Table 1:** The Baseline Characteristics of Patients with COVID-19

	Group A (n = 255)	Group B (n = 259)	*P value*
**Age (years)**	36.00 (29.00, 45.00)	34.00 (28.00, 45.00)	0.337
**Sex [n (%)]**			0.607
Male	60(23.53)	66(25.48)	
Female	195(76.47)	193(74.52)	
**Marital status [n (%)]**			0.257
Unmarried	45(17.6)	56(21.6)	
Married	210(82.4)	203(78.4)	
**Region [n (%)]**			**0.002**
North	20(7.8)	5(1.9)	
South	235(92.2)	254(98.1)	
**Residence [n (%)]**			0.979
Rural	41(16.1)	40(15.4)	
Township	67(26.3)	68(26.3)	
Urban	147(57.6)	151(58.3)	
**Medical staff [n (%)]**	153(60.0)	206(79.5)	**0.000**
**Smoking [n (%)]**			0.552
Never smoking	226(88.6)	231(89.2)	
Smoking	25(9.8)	21(8.1)	
Quit smoking	4(1.6)	7(2.7)	
**Drinking [n (%)]**			0.294
Never drinking	207(81.2)	210(81.1)	
Drinking	45(17.6)	41(15.8)	
Quit drinking	3(1.2)	8(3.1)	
**Physical Exercise [n (%)]**	99(38.8)	77(29.7)	**0.030**
**Sleeping Hours [n (%)]**			**0.001**
< 6 h	50(19.6)	82(31.7)^a^	
6–7 h	108(42.4)	110(42.5)	
≥7 h	97(38.0)	67(25.9)^a^	
**Bedtime [n (%)]**			**0.029**
10 pm and before 10 pm	65(25.5)	40(15.4)^a^	
11 pm	91(35.7)	112(43.2)	
12 pm	77(30.2)	88(34.0)	
after 12 pm	22(8.6)	19(7.3)	
**Disease History [n (%)]**	46(18.0)	54(20.8)	0.421
Hypertension [n (%)]	20 (7.8)	21 (8.1)	0.912
Diabetes [n (%)]	9 (3.5)	3 (1.2)	0.075
Hyperlipidemia [n (%)]	20 (7.8)	23 (8.9)	0.671
Chronic Respiratory Disease [n (%)]	3 (1.2)	3 (1.2)	0.985
Chronic Kidney Disease [n (%)]	3 (1.2)	1 (0.4)	0.308
Rheumatic Disease [n (%)]	5 (2.0)	3 (1.2)	0.462
Cancers [n (%)]	3 (1.2)	4 (1.5)	0.719
**Vaccination [n (%)]**			0.944
no	4(1.6)	4(1.5)	
one	3(1.2)	2(0.8)	
two	21(8.2)	24(9.3)	
three	212(83.1)	217(83.8)	
four	15(5.9)	12(4.6)	
**Treatment [n (%)]**			0.146
None	42 (16.5)	33 (12.7)	
Self-medication	200 (78.4)	203 (78.4)	
Medical prescription	13 (5.1)	23 (8.9)	

**Notes:**^a^Statistical comparison between group A and group B

Abbreviations: group A, the time for negative conversion < 11 days; group B, the time for negative conversion ≥11 days; COVID-19, coronavirus disease 2019

### Dietary patterns

The dietary data collected underwent exploratory factor analysis. The measure of sampling adequacy (MSA) of each factor is greater than 0.5. The Kaiser-Meyer-Olkin (KMO) test yielded a value of 0.73, and the Bartlett spherical test showed a significant result (c ²= 566.2, *P* < 0.001), further confirming the appropriateness of the data for factor analysis. When the number of common factors is four, the cumulative variance contribution rate is approximately 76.18%. Pattern A was responsible for 35.65% of the total variance, including cereals and starch, vegetables and fruits, and seafood. Pattern B accounted for 17.35% of the dietary intake variance and included beans and legumes, vegetables and fruits, and seafood. Pattern C, characterized by dairy products, eggs and related products, accounted for 12.44% of the total variance. Pattern D explained 10.75% of the dietary intake variance and encompassed meat and poultry, seafood, eggs and related products (Table 2). To further analyze the association between dietary patterns and negative conversion time, all participants were classified into tertiles based on their scores for each dietary pattern. The tertiles were labeled T1, T2, and T3, representing low, moderate, and high dietary pattern scores, respectively.

**Table 2:** Food group factor loading for mainly dietary patterns using factor analysis

Groups	Pattern A	Pattern B	Pattern C	Pattern D
Cereals and starch	0.938	−	−	−
Vegetables and fruits	0.531	0.480	−	−
Meat and poultry	−	−	−	0.905
Milk and dairy products	−	−	0.792	−
Seafood	0.472	0.378	−	0.404
Eggs and related products	−	−	0.783	0.349
Beans and legumes	−	0.933	−	−
Variance explained (%)	35.65	17.35	12.44	10.75

**Notes:** groups with factor loadings ≥0.3 and ≤ -0.3

### Association between age and negative conversion time of patients with COVID-19

**Fig. 3:**
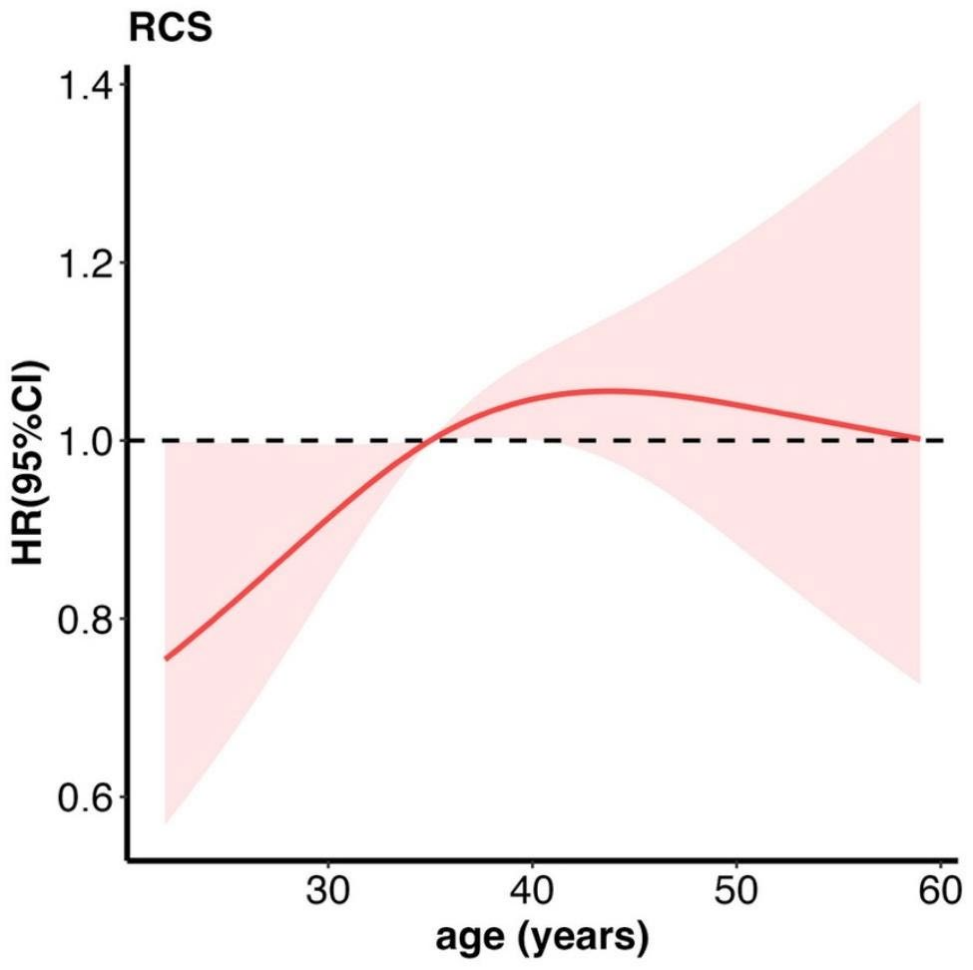
Hazard ratio function for age to negative conversion rate. **Abbreviations**: RCS, restricted cubic splines; HR, hazard ratio; CI, confidence interval

We used restricted cubic splines to model the relationship between age and negative conversion time. Figure 3 showed that the association between age and negative conversion rate followed an inverted U-shaped pattern. Specifically, for individuals below 35 years of age, there was a negative association between age and the hazard ratio for negative conversion rates. Conversely, for individuals above 35 years, a positive association was observed. Figure 2 illustrated that the hazard ratio of negative conversion rates increased with age until approximately 45 years, after which it declined. Based on these findings, we divided age into three categories: <35 years, 35–45 years, and ≥ 45 years.

### Association between risk factors and negative conversion time of patients with COVID-19 among different age groups

**Fig. 4:**
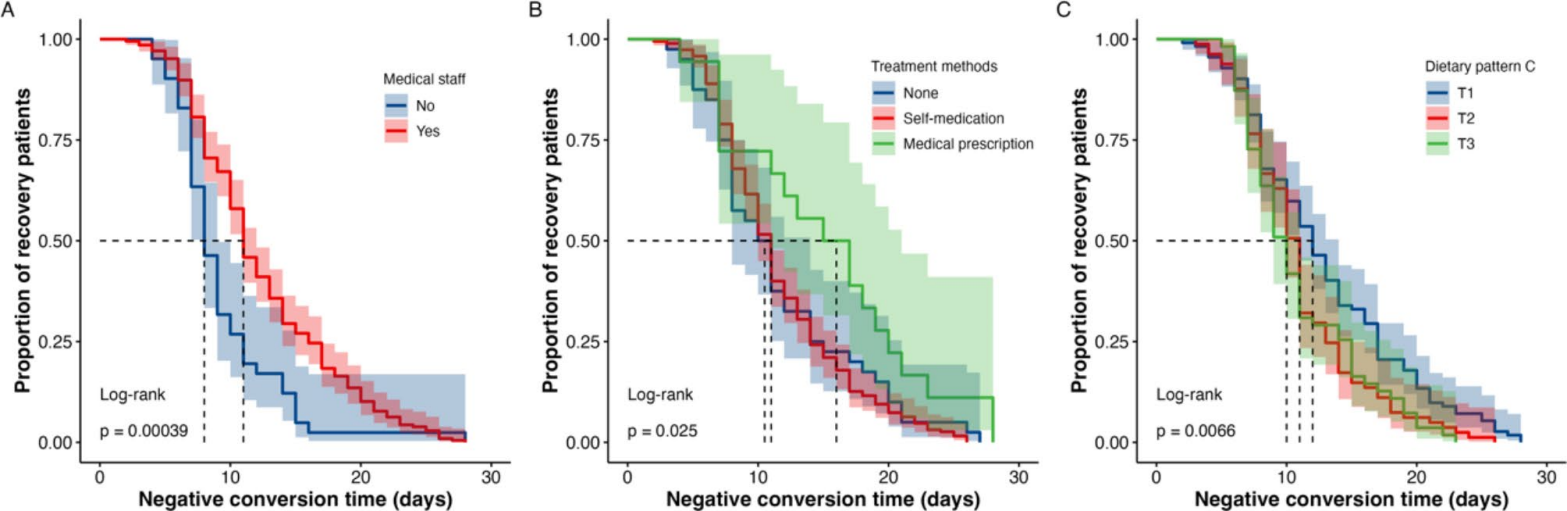
Kaplan-Meier plot of medical staff (**A**), treatment methods (**B**), dietary pattern C(**C**) and negative conversion time in patients infected COVID-19 under 35 years old. **Notes**: Dietary pattern C was characterized by milk and dairy products, eggs and related products. **Abbreviations**: T, tertiles

**Fig. 5:**
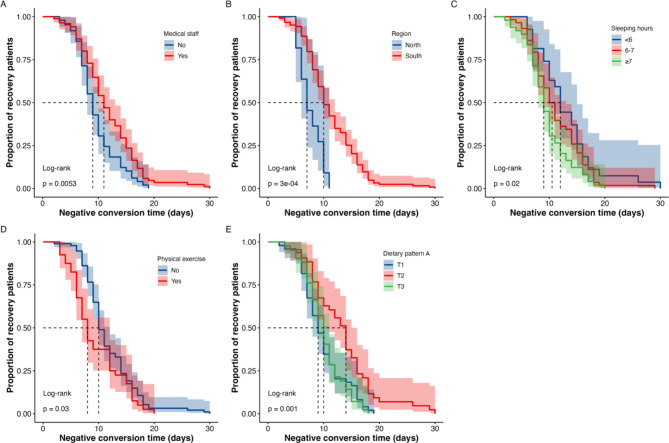
Kaplan-Meier plot of medical staff (**A**), region (**B**), sleeping hours (**C**), physical exercise (**D**), dietary pattern A (**E**) and negative conversion time in patients infected COVID-19 aged 35 ~ 45 years. **Notes**: Dietary Pattern A including cereals and starch, vegetables and fruits, and seafood. **Abbreviations**: T, tertiles

**Fig. 6:**
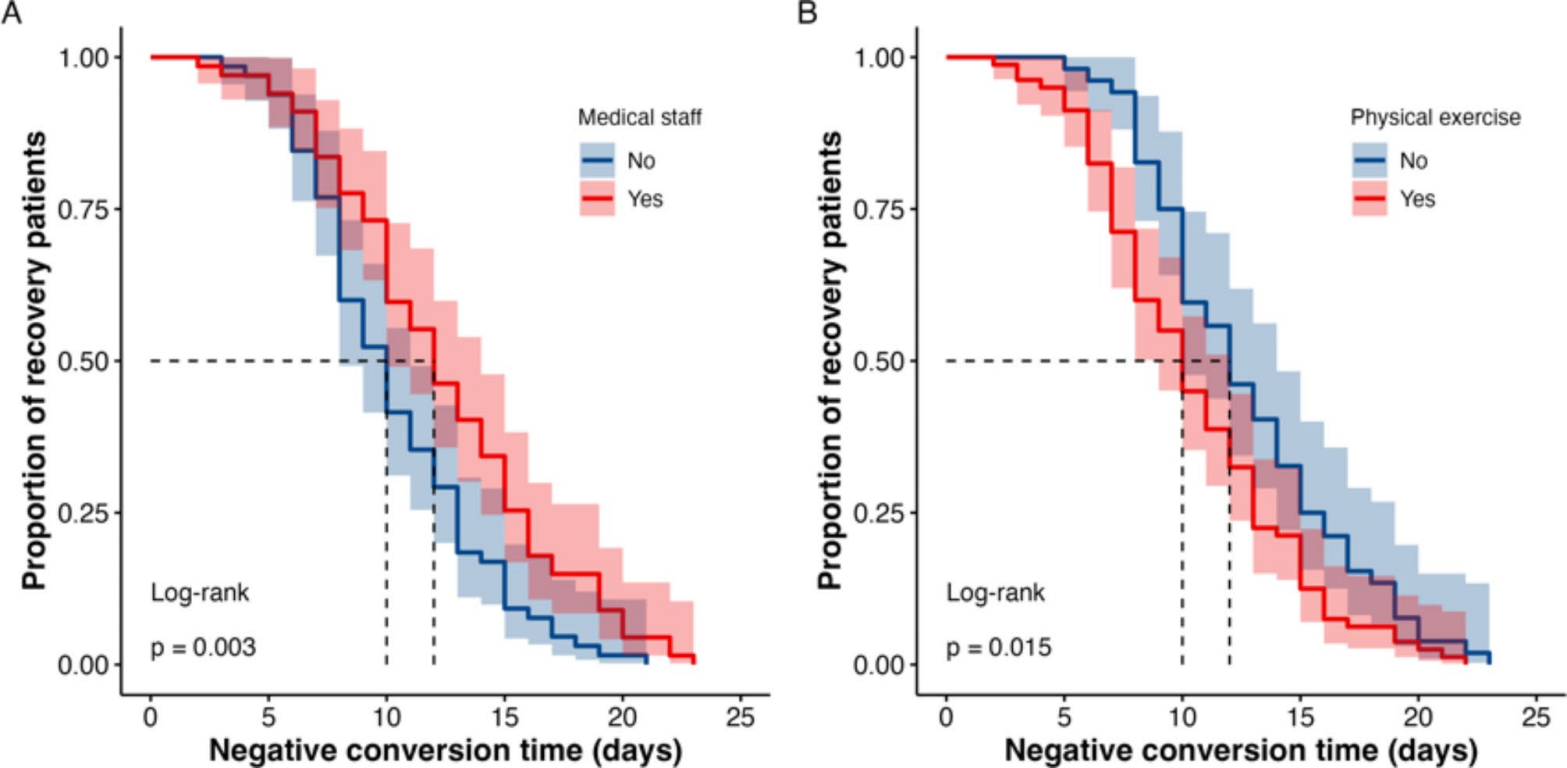
Kaplan-Meier plot of medical staff (**A**), physical exercise (**B**) and negative conversion time in patients infected COVID-19 aged 45 years or older

Based on the above age groups, the Kaplan-Meier curve and Cox regression analysis were used to analyze the differences among different age groups. In the population under 35 years old, both the Kaplan-Meier plot and Cox regression analysis indicated that a higher intake of dietary pattern C (rich in protein) was associated with a faster recovery among COVID-19 patients, whereas medical staff or participants receiving prescription treatments exhibited a slower recovery rate (all *P* < 0.05; Table 3; Fig. 4). In the population aged 35–45, the Kaplan-Meier plot revealed that COVID-19 patients who were medical staff or resided in the south or had a higher intake of dietary pattern A (rich in carbohydrates) experienced a longer negative conversion time, while those who engaged in regular exercise or had sufficient sleep exhibited a shorter negative conversion time (all *P* < 0.05; Fig. 5). Cox regression results showed that sufficient sleep and physical exercise were associated with a shorter time to negative conversion, while individuals from southern regions and those with a higher intake of dietary pattern A (rich in carbohydrates) had a longer time to negative conversion (*P* < 0.05; Table 3). In the population aged 45 years or older, the Kaplan-Meier plot and multivariate Cox regression analysis suggested the time of COVID-19 negative conversion was only related to physical exercise and being a medical staff member, the correlation between sufficient sleep and negative conversion time was only evident in univariate Cox regression result (*P* < 0.05; Table 3; Fig. 6).

**Table 3:** Univariate and multivariate Cox regression analyses of risk factors for negative conversion time

	Univariate analysis				Multivariate analysis			
	HR	95%CI	*P* value		HR	95%CI	*P* value	
< 35 years								
**Medical staff (no vs. yes)**	0.55	(0.39, 0.78)	**< 0.001**		0.53	(0.39, 0.76)	**< 0.001**	
**Dietary pattern C**								
T1	Reference				Reference			
T2	1.49	(1.11, 1.99)	**< 0.01**		1.35	(1.00, 1.81)	**< 0.05**	
T3	1.55	(1.12, 2.16)	**< 0.01**		1.40	(1.00, 1.97)	**< 0.05**	
**Treatment**								
None	Reference				Reference			
Self-medication	1.07	(0.76, 1.51)	0.703		1.01	(0.71, 1.43)	0.95	
Medical prescription	0.53	(0.30, 0.95)	**< 0.05**		0.48	(0.26, 0.86)	**< 0.05**	
**35 ~ 45 years**								
**Region (north vs. south)**	0.31	(0.16, 0.59)	**< 0.001**		0.46	(0.23, 0.93)	**< 0.05**	
**Medical staff (no vs. yes)**	0.60	(0.42, 0.86)	**< 0.01**		0.74	(0.51, 1.08)	0.120	
**Sleeping hours**								
< 6 h	Reference				Reference			
6–7 h	1.35	(0.85, 2.15)	0.279		1.38	(0.86, 2.22)	0.176	
≥7 h	1.92	(1.18, 3.11)	**< 0.01**		1.79	(1.09, 2.95)	**< 0.05**	
**Physical exercise (no vs. yes)**	1.47	(1.01, 2.14)	**< 0.05**		1.48	(1.02, 2.17)	**< 0.05**	
**Dietary pattern A**								
T1	Reference				Reference			
T2	0.50	(0.33, 0.78)	**< 0.01**		0.50	(0.32, 0.78)	**< 0.01**	
T3	1.04	(0.69, 1.58)	0.840		0.91	(0.59, 1.40)	0.661	
**≥ 45 years**								
**Medical staff (no vs. yes)**	0.59	(0.42, 0.84)	**< 0.01**		0.62	(0.44, 0.89)	**< 0.01**	
**Physical exercise (no vs. yes)**	1.53	(1.07, 2.19)	**< 0.05**		1.48	(1.03, 2.11)	**< 0.05**	
**Sleeping hours**								
< 6 h	Reference				Reference			
6–7 h	1.40	(0.93, 2.10)	0.110		1.38	(0.91, 2.09)	0.223	
≥7 h	1.56	(1.00, 2.42)	**< 0.05**		1.46	(0.94, 2.28)	0.092	

**Notes:** Dietary Pattern A including cereals and starch, vegetables and fruits, and seafood. Dietary pattern C was characterized by milk and dairy products, eggs and related products

**Abbreviations:** HR, hazard ratio; CI, confidence interval; T, tertiles

## Discussion

Coronavirus disease 2019 (COVID-19) is a global public health event characterized by its rapid transmission, wide infection range and high difficulty in prevention and control both in China and worldwide [20]. Considering these factors, it is crucial to actively seek effective measures to alleviate the symptoms of COVID-19 and reduce the time required for negative conversion. This study conducted a retrospective analyzed of the lifestyle and general characteristics of 514 patients who were infected with COVID-19 after December 2022 and had achieved negative conversion. Our study revealed there was a negative correlation between age and risk of negative conversion for COVID-19 in the age group of 18–35, indicating that younger individuals experienced a longer duration of negative conversion, which contrasts with the results observed in individuals older than 35 years. This result was not entirely consistent with previous studies. A retrospective cohort study of adult patients with non-severe COVID-19 at Eastern China Hospital showed that older age was a risk factor for persistent infection with the Omicron mutation beyond 14 days [21]. Mo P et al [22] reported that patients with long-term conversion were older compared to those with short-term negative conversion. This result can be explained by the fact that older individuals often experience a compromised immune system and a higher occurrence of pre-existing health conditions. Since the study questionnaire was conducted through WeChat Moments, the age of our study subjects tended to be relatively young. The median age was 35 years (ranged from 20 to 78 years), and only 8 patients (1.6%) were elderly (≥ 60 years), which results in a lower prevalence of underlying health conditions. Consequently, Consequently, we did not observe a correlation between age, underlying health conditions and negative transition time for COVID-19. Based on the above findings, we categorized age into three categories: <35 years, 35–45 years, and ≥ 45 years and looked for risk factors within each group. Because of occupational exposure and inadequate rest, medical staff experienced a significantly longer duration for negative transformation. Our study indicated that medical staff in our study experienced a slower recovery compared to other participants in different age groups.

Fermented dairy products are known to be natural ACE inhibitors. Previous studies have shown that the consumption of higher fermented dairy products can reduce the severity and mortality of COVID-19 [23, 24]. In this study, we used factor analysis to establish four dietary patterns. Our study demonstrated that a high intake of dairy products had a positive impact on the negative conversion of COVID-19 in population under 35 years old. However, in the population aged 35 ~ 45 years, higher carbohydrates intake could delay the negative conversion. While previous studies and recommendations have focused on the benefits of vitamins, minerals, and other nutrients for patients with COVID-19 [24, 25], limited studies examined the impact of carbohydrates on COVID-19 patients. The sample size of this study is small and further confirmation through large-scale research is needed.

During physical exercise, muscle and adipose tissue produce a substance called irisin, which decreases the expression levels of TLR3, TLR4, MyD88, MAPK, and NF-kB, thereby reducing the release of pro-inflammatory factors and preventing the development of sepsis [26]. In addition, irisin can also improve pro-inflammatory status by reducing the activation of mTOR and NF-kB [27, 28]. Our study confirmed that the proportion of individuals engaging in physical exercise was significantly higher in the group with a shorter duration of negative conversion time compared to the group with a longer duration. Both KM plots and COX regression analysis indicated that exercise may shorten the duration of negative conversion in patients with COVID-19 in individuals over 35 years old. A prospective cohort study found that lack of physical exercise increased the risk of COVID-19 hospitalization in adults by 32% [29]. Another study revealed that COVID-19 patients who remained physical inactive had a 126% higher risk of hospitalization compared to those who consistently met physical activity guidelines [30]. However, Nieman DC et al [31] confirmed that sustained and intensive exercise could trigger widespread immune disturbances, accompanied by increased inflammation, oxidative stress, leading to an increased risk of certain diseases. The American College of Sports Medicine (ACSM) encouraged health individuals to engage in moderate physical activity for 150 to 300 min per week, but avoid overtraining if they have a significant risk of SARS-CoV-2 exposure [32]. Therefore, it is important to maintain regular physical exercise of moderate intensity. Nevertheless, if individuals are infected with COVID-19, it is advised to reduce the intensity of exercise and seek guidance from healthcare professionals.

Previous studies demonstrated that people with shorter sleep duration, sleep disorders, or poor sleep quality were more likely to contract respiratory diseases [33–36]. Bjorvatn B et al [37] found that once infected coronavirus, shift/night shift workers were more likely to experience symptoms exceeding 8 weeks, with a 171% increased risk of developing from mild to moderate or critical illness, as well as a 466% increased probability of needing hospital treatment. In line with previous studies, our study indicated that the group getting 7h or more hours of sleep had a higher number of individuals with a shorter negative conversion duration. Additionally, among those who going to bed at 9 pm or earlier, individuals with shorter negative conversion times had higher rates compared to those with longer durations. Disruption of the circadian rhythm and sleep increases the production of proinflammatory cytokines and puts the body in a state of low-functioning immune function, thus making the body more susceptible to disease [38]. Moreover, a substance that can reduce pro-inflammatory cytokines s like TNF-a and IL-6 while increasing anti-inflammatory cytokines such as IL-10 [39], is more abundant during the night and less during the day. In addition, melatonin has crucial antioxidant properties that effectively prevent cell damage and reduce tissue damage caused by inflammation [40]. This hormone can further affect the immune response by stimulating the proliferation and maturation of various specialized cells, such as NK cells, T and B lymphocytes, granulocytes, and monocytes, thereby enhancing adaptive immunity [41].

Previous studies have mostly focused on the experts’ recommendations regarding the lifestyle of COVID-19 patients or the influence of lifestyle on the severity of the disease. Our study is the first to assess the impact of various lifestyles factors, including sleep, physical exercise, dietary patterns and more on the time of negative conversion for COVID-19 patients. This research provides valuable insights for offering improved lifestyle guidance to individuals dealing with COVID-19 and other diseases. However, there are some limitations in our study. First, there is a certain degree of selection bias due to the relatively small proportion of individuals who smoke, drink, are unvaccinated, or are elderly in our study. This may limit the generalizability of our findings to broader populations. Secondly, the study had a large number of medical staff, but no specific occupational groups were compared. Thirdly, the lack of biochemical indicators such as vitamin D and NLR limits our comprehensive assessment of risk factors for COVID-19 to achieve negative conversion. Finally, the small sample size of our study needs confirmation by a larger sample size to enhance the reliability and validity of the results.

## Conclusions

In summary, our study findings indicate distinct associations between lifestyle factors and the duration of negative conversion in COVID-19 patients among different age groups. The adoption of healthy lifestyle, such as maintaining adequate sleep, regular physical exercise, and increasing protein intake in our daily diet, can enhance our resilience and contribute to disease alleviation. Conversely, being a medical staff member or having excessive carbohydrates intake were associated with longer duration for negative conversion.

## Data Availability

The data that support the findings of this study are available from the corresponding author on reasonable request but restrictions apply to the availability of these data, which were used under license for the current study, and so are not publicly available.
